# The Blood-Stained Pillow

**Published:** 1900-06-30

**Authors:** 


### THE BLOOD-STAINED PILLOW.

At the last meeting of the Royal Medical and Chirurgical Society a very interesting discussion arose as to the condition known as pyorrhœa alveolaris, commonly spoken of as "spongy gums." To some this may have appeared to be a mere local affair of small importance, but observations are not wanting to show that the continued ingestion of a more or less purulent secretion is distinctly detrimental to the economy. Mr. Godlee, in the paper which he read on the subject, stated that, although not usually the cause of serious trouble, pyorrhœa alveolaris might give rise to symptoms which simulated those of graver disorders. The disease consisted of a spongy condition and recession of the gums, accompanied, and perhaps caused, by the deposit of tartar round the teeth. The inflammation extended to the peridental membrane or periosteum of the fang, and the consequence in some cases was that pockets of pus were formed between this membrane and the tooth. The disease often appeared to be hereditary, and was very chronic and difficult of cure. Four examples were given in which conditions not at first sight apparently connected with the state of the gums showed remarkable improvement when the gums had been effectually treated. Two of them were cases of pleurisy, suggesting the suspicion of there being a basic cavity, the third was that of a man, aged 66, affected by the disease in a marked degree, who was attacked with acute glossitis and stomatitis, with diarrhœa and pyrexia, while in the fourth case the symptoms were those of cancer of the stomach. In all these cases, after treatment directed to the gums, the symptoms disappeared. It seems pretty clear, however, that the cases brought forward by no means cover the field, and that the disease has even more wide-reaching consequences than those mentioned by Mr. Godlee. One need, in fact, hardly be surprised to find that the frequently-repeated ingestion of inflammatory products, wherever they come from, produces injurious effects. Nor can one omit in this connection to refer to the researches of Dr. William Hunter, of Charing Cross Hospital, in regard to the origin of pernicious anæmia from an infection arising within the mouth. He points out the frequency with which an unusually cario-necrotic condition of the teeth is met with as an antecedent in pernicious anæmia, and he draws the conclusion that the origin of the infection underlying pernicious anæmia is in most cases to be traced to the teeth. The subject is well worthy of the most careful investigation. The mouth is so admirable a breeding ground for so many bacterial forms, it is so open to infection from without, and the products resulting from bacterial growth are so sure to be absorbed, that we need hardly be surprised to find that people suffering from chronic inflammation of the gums, with pockets of pus between them and the teeth, suffer also from various chronic forms of toxæmia. Yet, without special inquiry, this cause of disease may not be discovered, for so used do patients become to their condition that they may not complain about it, and there may be little sign of its presence except the blood-stained pillow.

